# PALM: A Paralleled and Integrated Framework for Phylogenetic Inference with Automatic Likelihood Model Selectors

**DOI:** 10.1371/journal.pone.0008116

**Published:** 2009-12-07

**Authors:** Shu-Hwa Chen, Sheng-Yao Su, Chen-Zen Lo, Kuei-Hsien Chen, Teng-Jay Huang, Bo-Han Kuo, Chung-Yen Lin

**Affiliations:** 1 Institute of Information Science, Academia Sinica, Taipei, Taiwan; 2 Division of Biostatistics and Bioinformatics, National Health Research Institutes, Zhunan, Miaoli County, Taiwan; 3 Institute of Fishery Science, College of Life Science, National Taiwan University, Taipei, Taiwan; 4 Research Center of Information Technology Innovation, Academia Sinica, Taipei, Taiwan; University of Manchester, United Kingdom

## Abstract

**Background:**

Selecting an appropriate substitution model and deriving a tree topology for a given sequence set are essential in phylogenetic analysis. However, such time consuming, computationally intensive tasks rely on knowledge of substitution model theories and related expertise to run through all possible combinations of several separate programs. To ensure a thorough and efficient analysis and avert tedious manipulations of various programs, this work presents an intuitive framework, the phylogenetic reconstruction with automatic likelihood model selectors (PALM), with convincing, updated algorithms and a best-fit model selection mechanism for seamless phylogenetic analysis.

**Methodology:**

As an integrated framework of ClustalW, PhyML, MODELTEST, ProtTest, and several in-house programs, PALM evaluates the fitness of 56 substitution models for nucleotide sequences and 112 substitution models for protein sequences with scores in various criteria. The input for PALM can be either sequences in FASTA format or a sequence alignment file in PHYLIP format. To accelerate the computing of maximum likelihood and bootstrapping, this work integrates MPICH2/PhyML, *PalmMonitor* and *Palm job controller* across several machines with multiple processors and adopts the task parallelism approach. Moreover, an intuitive and interactive web component, *PalmTree*, is developed for displaying and operating the output tree with options of tree rooting, branches swapping, viewing the branch length values, and viewing bootstrapping score, as well as removing nodes to restart analysis iteratively.

**Significance:**

The workflow of PALM is straightforward and coherent. Via a succinct, user-friendly interface, researchers unfamiliar with phylogenetic analysis can easily use this server to submit sequences, retrieve the output, and re-submit a job based on a previous result if some sequences are to be deleted or added for phylogenetic reconstruction. PALM results in an inference of phylogenetic relationship not only by vanquishing the computation difficulty of ML methods but also providing statistic methods for model selection and bootstrapping. The proposed approach can reduce calculation time, which is particularly relevant when querying a large data set. PALM can be accessed online at http://palm.iis.sinica.edu.tw.

## Introduction

Advances in molecular biology and bioinformatics have enabled researchers to obtain gene sequences by experimental procedures, as well as by sequence searching approaches. The feasibility of utilizing sequence features that can be viewed as evolutionary changes in the sequence occurring in the operating taxonomic units has received considerable interest. A molecular phylogenetic analysis procedure based on sequence features is initiated by gathering a set of sequences derived from a common origin. Corresponding residues among DNA/protein sequences are then defined by multiple sequence alignment (MSA). Next, the relatedness among the input sequences is estimated using the phylogenetic inference method with a suitable substitution model. Finally, the representative tree(s) of the phylogenetic relationship is constructed and may be presented graphically with statistical confidence of the branching topology. Significant advances in theoretical and mathematical implementation of phylogenetic methodology have been made in recent decades. These methodological advances provide an unprecedented and often bewildering set of choices on methodological issues [1]. Researchers unfamiliar with phylogenetic analysis are perplexed by selecting and installing programs, transferring files between programs, setting parameters for the complete process, running iterations with various combinations of methods and parameters [2], as well as recalling all tools and parameter-related choices made during analysis. Although the relationship among an interesting sequence set from an evolutional aspect has received considerable attention, performing adequate phylogenetic analysis with a sound theoretical foundation is quite difficult.

Among the three major categories of phylogenetic inference methods, distance, maximum parsimony (MP), and maximum likelihood (ML), ML methods are especially useful for sequence sets with varying extents of sequence diversity [2], [3]. Based on the theory of ML methods, the likelihood of a series of residue substitution events is estimated and, then, the most probable tree topology from all possible ones that represents the evolutionary history of a given sequence set can be inferred. Various residue substitution models that describe the probability of replacing one residue with another have been derived from either statistical analysis involving conserved sequence blocks or molecular evolution theories [4], [5], and can be further refined for parameters involving sites under the selection force or for varying substitution rates among relevant sites. Likelihood-based approaches are robust for phylogenetic inference. Although obtaining a best-fit ML tree with an increasing number of sequences and sequence length is computationally intractable (NP-hard), a ML-based method can provide statistical comparability of the fitness of substitution models [6], [7].

There are several ML-based phylogenetic inference programs available, including PhyML [8], [9], PAML [10], [11], Multiphyl [12], Phylip package [13] and IQPNNI [14]. Owing to a heavy computational load, several attempts have been made to accelerate the estimation of ML by refining algorithms and applying parallel computing, e.g., PAML [10], [11], RAxML [15], GARLI [16], mpi-CH2/PhyML [9] and pIQPNNI [17]. These programs are performed in command-line mode or in a graphical-user interface. Some of the phylogenetic servers, such as PhyML web server [9], Multiphyl web server [12], phylogeny.fr [18] and Phylemon [19], are available, providing computational power and a user-friendly interface. Users should normally be aware of phylogenetic analysis, and then achieve a reasonable outcome from these web applications.

How to select an optimal substitution model for ML estimation is addressed in [20]. The implementations of best model selection procedures were proposed for both protein sequences [21] and nucleotide sequences [22]. ProtTest [21] is a model selection scheme for protein sequences based on score files generated by PhyML. The fitness of amino acid substitution models is estimated using three scores, i.e. Akaike information criterion (AIC), corrected AIC (AICc) and Bayesian Information Criterion (BIC), as well as the maximum likelihood of each model. Modeltest [22] applies AIC, AICc, BIC, and hierarchical likelihood ratio tests (hLRTs), to estimate the model fitness from the score file of DNA substitution models generated by PAUP [23]. Currently, MODELTEST has been superseded by jModeltest [24], an integrated framework using PhyML [8] computational procedures to provide the nucleotide substitution model selection. jModeltest provides two additional evaluation criteria, i.e. dynamical likelihood ratio tests (dLRT) and a decision theory method (DT), as estimates of model selection uncertainty, parameter importance and model-averaged parameter estimates, including model-averaged phylogenies. FINDMODEL [25] implemented the idea of MODELTEST for testing the best fit model for the input alignment of nucleotide sequences. FINDMODEL provides options of selecting the model set and tree methods for constructing the initial tree. The best substitution model from 28 models is determined by the lowest AIC of the optimized ML tree topology, as estimated by baseml (PAML) and MODELTEST. The model estimators described above take aligned sequences as input and select the most appropriate model based on the probability (the likelihood) of the ML tree. Once the model has been set, a ML-based phylogenetic inference program can be applied for bootstrapping and the possible phylogenetic relationship presented in a tree topology obtained as well. The entire process, from the model selection to bootstrapping, is time consuming and computationally intractable, especially when handling large data sets.

To perform comprehensive phylogenetic analysis with a model selection mechanism without monotonous manipulations of data input/output, this work describes the design of an intuitive framework for phylogenetic reconstruction with automatic likelihood model selectors (PALM). By using the proposed integrated framework of ClustalW, PhyML, MODELTEST, ProtTest, and several in-house programs (*PalmDaemon*, *PalmMonitor*, *PalmTree*, and *Palm job controller*), the fitness of 56 substitution models is evaluated for nucleotide sequences and 112 substitution models for protein sequences with scores in various criteria (*i.e.* AIC, AICc, BIC and hLRT). Via the parallel computing strategy, the calculation time of this ML-based phylogenetic reconstruction tool is substantially reduced. All parameters used and outputs generated by each program can be accessed online. Moreover, resubmitting a new job from previous graphic results to remove rogue taxa and add new ones is an effortless task when using *PalmTree*.

## Methods

### Structure of PALM System

This work adopts the maximum likelihood (ML) method, which facilitates the application of mathematical models that incorporate the knowledge of typical patterns of sequence evolution, resulting in more powerful phylogenetic inferences [7]. To ensure a stable system with satisfactory performance, PALM is constructed on a platform with several symmetrical multi-processor (SMP) servers equipped with quad CPUs (700MHz Intel Xeon) and 8 GB RAM. Additionally, the LAMP structure (Ubuntu, version 8.04; Apache, version 2.04; Postgresql, version 8.3.7; PHP, version 5.1.0) and a MS-Window 2003 server are adopted to take the computation load and streamline the workflow control (*PalmDaemon*).

**Figure 1:**
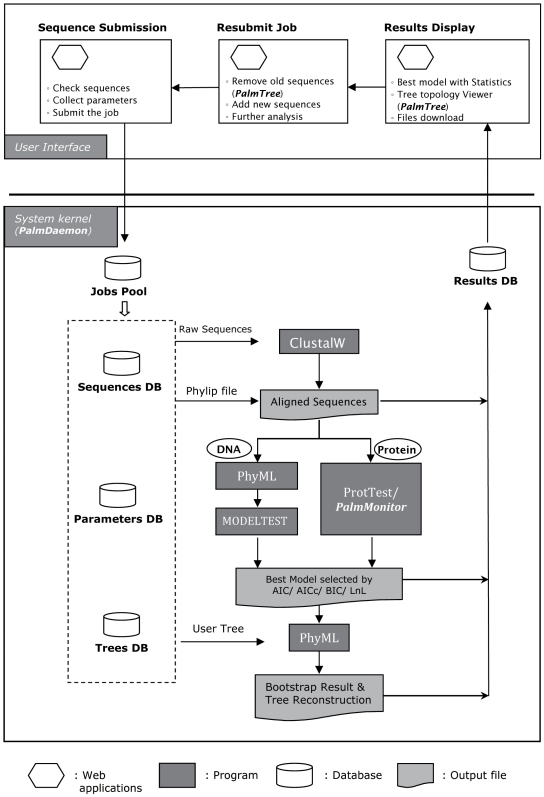
Infrastructure and workflow of PALM.

PALM is designed for biologists without prerequisite computer skills to run a complex phylogenetic analysis process via a succinct, user-friendly interface. Achieving this objectives involves integrating several well-established programs (*i.e.* readseq [26] version 2.1.26, ClustalW [27] version 2.0.1, PhyML [8] version 3.0, MODELTEST [22] version 3.7, and ProtTest [21] version 2.0) with our applications, including *PalmDaemon*, *PalmMonitor*, *PalmTree* and *Palm job controller* into a seamless pipeline (Fig. 1).

### *PalmDaemon*

Based on the design of PALM, a series of successive calculation steps is transformed into an integrated service. *PalmDaemon* is responsible for transferring data between programs and agglutinating several prestigious programs and our in-house programs. Following selection of the optimum model, the alignment, parameters for model choice, and bootstrap setting are submitted to *Palm job controller* for parallel computing on bootstrapping. To easily retrieve the output results, *PalmDaemon* sends e-mails notifying job acceptance and job completion to users. Following the job ID link in the notification mail, users can trace back to their job results. Query parameters are stored and used if a resubmission job is initiated from the *PalmTree* Viewer (as described below). Additionally, to avert long sequence identifier (ID) truncation caused by alignment, the sequence IDs (up to 100 characters) are converted into internal running IDs by *PalmDaemon* and are restored in the final outputs. Therefore, meaningful sequence IDs encoded with a message such as the sampling date and species name are allowed, subsequently making the result comprehensible.

### *PalmMonitor*: Multi-Thread Processing

PALM runs through tree-building processes of all available models and then identifies the most appropriate model in terms of the tree with the best likelihood -derived score by the model. This time consuming task is accelerated by implementing a multi-thread dispatch program in Java, *PalmMonitor*. *PalmMonitor* is used to divide a single sequential task of running 56 nucleic acids substitution models and 112 protein substitution models into parallel ones and, then, merges the results when all tasks are completed. Doing so reduces a considerable amount of computational time, *i.e.* a six-threading run reduces the computing time from eight hours to eighty minutes for a query of 20 sequences with 5000 residues in length.

### *PalmTree*: An Interactive Tree Viewer and a Job Resubmission Gateway

*PalmTree* is a versatile tool composed of C, php and Java languages for viewing the tree topology and restarting analysis. *PalmTree* parses the context of the Newick format tree file and converts the text layout into a graph. In this work, the tree topology is provided by one of three options, *i.e.* the original tree plot, unrooted tree, and pseudo-rooted tree by the mid-point method [28]. The mid-point method is adopted as the default presentation of tree topology; the mid-point of the longest node-to-node path is set as an artificial rooting node and a pseudo-rooted tree in a balanced structure is displayed. The branch length and the bootstrapping value (in percentage) in the output tree file are optional display parameters. Additionally, the tree branch edge can be broadened according to the bootstrapping value, thus providing a direct impression on the confidence on the branching pattern. User can click on the branching point to perform the branch swapping without altering the tree topology. Notably, any leaf node, *i.e.* a sequence in the original query, can be removed from the tree effortlessly with a mouse click; meanwhile, undeleted sequences can be re-submitted to PALM for analysis. Additionally, new sequences can be added through the job submission form for reinitiating the analysis.

### *Palm Job Controller*: A Paralleled Task Controller for Bootstrapping

An attempt is made to enhance the performance of PALM by conducting distributed computing in a remote python call (RPyC, http://rpyc.wikidot.com/), a transparent and symmetrical python library for remote procedure calls, clustering and distributed-computing, with mpi-PhyML. Based on this framework, multiple submissions can simultaneously be processed dynamically to overcome the physical boundaries between processers and computers. This feature implies that PALM can modify the thread from one to many in order to avoid a computationally burdensome task from occupying the entire system.

## Results

### 1. PALM Workflow

PALM analysis begins by submitting a selected sequence set or pre-aligned sequences. Submitted nucleic acid sequences or protein sequences in FASTA format are computed for the output alignment in PHYLIP format using ClustalW; a query initiated in a pre-aligned file in PHYLIP format by other sequence align tools disregards this procedure. The alignment is then transferred to an automatic model selecting process in *PalmDaemon* and *PalmMonitor* by routing through PhyML/MODELTEST for DNA and PhyML/ProtTest for protein sequences, respectively. The fitness among 56 substitution models (JC69, K80, F81, HKY, TrN, TrNef, K3P, K3Puf, TIM, TIMef, TVM, TVMef, SYM, and GTR with parameter G, I) for nucleotide sequences and 112 models (LG, DCMut, JTT, MtREV, MtMam, MtArt, Dayhoff, WAG, RtREV, CpREV, Blosum62, VT, HIVb, and HIVw with parameter I, G, F) for protein sequences is estimated in PALM, and the optimal model are used to infer the phylogeny. The models denoted by +I imply that a fraction of data set is assumed to be invariable, where +G is considered to categorize the change of substitution rates among sites in discrete gamma distribution. Besides, +F implies that the equilibrium base frequencies in the sequences are estimated by observing the occurrence in the data.

Once the maximum likelihood for all available models is estimated and merged into a file by *PalmDaemon*, statistical information of the parameters can be accessed to evaluate the fitness of a model for a given alignment. In this work, three criteria are provided, *i.e.* Akaike Information Criterion (AIC, and the derived AICc and AICw), Bayesian Information Criterion (BIC) and the maximum likelihood value (LnL), as customized options for ranking the fitness of models. Finally, the top ranked model is selected for the phylogenetic inference with iterations via PhyML, and a tree topology with a bootstrap value is generated. The user is notified via e-mail with a link to the PALM result page after the task is completed (Fig. 1).

### 2. PALM Output

**Figure 2:**
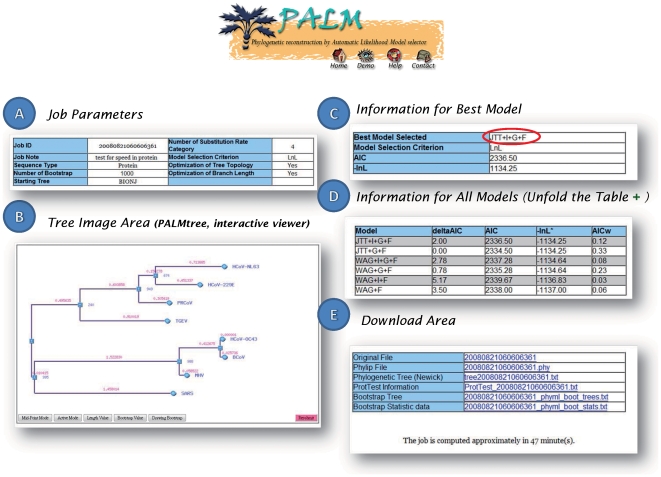
PALM Output. The result page consists of five parts. A). Job parameters. The job ID and user-defined parameters in the submission are included. B). Tree topology drawn by *PalmTree*, an interactive topology viewer with displaying options of bootstrapping value and branch length. A mouse click on a branching point can make the sub-tree flip; a click on the end node (round, with sequence ID) removes the sequence from the submitted data set before reinitiating an analysis procedure. C). Information about the best model selected by *PalmDaemon*. D). Statistics on all models calculated by *PalmDaemon*. E). Download area for those files generated from the entire PALM process.

Figure 2 summarizes the results of PALM, as categorized in five parts. **Job parameters** refer to submission-related information, including a job ID, sequence type, bootstrap number, model selection criterion and number of substitution rate categories. A **tree image area** is the graph output from *PalmTree*. **Information for the best model** describes the summary of the model being selected for the phylogenetic inference. An expendable, **information for all model** tables displays the details of the maximum likelihood for models sorted by the ranking option set for model selection. All output files generated during the reconstruction process are available in the **download area**. Moreover, PALM provides a mechanism to reinitiate the analysis for a user to add and remove sequences on each experiment via *PalmTree* (tree image area), as mentioned earlier. This mechanism allows users to identify inappropriate/irrelevant sequences and delete sequences in a visualized environment.

## Discussion

PALM is an integrated framework of ClustalW, PhyML, MODELTEST, ProtTest and several in-house programs to evaluate the fitness of 56 substitution models for nucleotide sequences and 112 substitution models for protein sequences with scores in various criteria. It is especially useful for biologists to perform phylogenetic analyses without prerequisite computer skills. Users are free of tedious tasks of sequence/file format conversion. Problems incurred by sequence alignment on long descriptions as sequence identifiers are also resolved. Hence, the sequence IDs can be displayed correctly for telling biological truths in both the tree topology and in the newick format tree file. In contrast with three other renowned phylogenetic web servers, Phylogeny.fr [18], Phylemon [19] and Multiphyl [12], the entire workflow of PALM is straightforward and coherent tightly from the job submission to the output retrieving with even more efficiency and more available substitution models. Unlike Phylogeny.fr, PALM does not allow users to define their choice stepwise. For users who prefer to set all their choices, a complementary action to PALM can be performed in our previous work, POWER (PhylOgenetic WEb Repeater, http://power.nhri.org.tw) [29] which is designed for running the phylo- genetic inference based on Phylip package (http://evolution.gs.washington.edu/phylip.html) for specific methods of phylogenetic inference (mainly in the distance method, maximum parsimony, and some maximum likelihood method). Otherwise, users may execute their request on the expert mode of phylogeny.fr or phylemon for ML methods with phylogeny knowledge.

There are several MSA tools available, including ClustalW and ClustalX [27], MUSCLE [30] MAFFT [31], DIALIGN [32], [33], and T-coffee [34]. Essoussi *et al.* compared several alignment tools, indicating that no single tool can consistently outperform other ones by the estimation of quality alignment [35]. This work thus incorporates ClustalW as the built-in alignment tool of PALM owing to its stable performance and popularity. Meanwhile, pre-aligned sequences in PHYLIP format can be accepted by PALM as input to fulfill user requests with respect to alignment from various perspectives.

As mentioned earlier, a ML-based method is computationally difficult. After the number of input sequences, or the sequence length, or the number of available models is increased, the computing task of ML burdens the system. For instance, jModeltest [24] was designed to estimate the optimal model starting from aligned sequences. A large dataset ran in jModeltest is time consuming (*e.g.*, 4 hours for 24 sequences on an average of 4600 bps). Smoothly implementing the system for research purposes necessitates setting PALM in a reasonable running time for a job query. For accelerating ML calculations, this work presents a novel workflow framework by integrating PhyML, MODELTEST, *PalmMonitor* and the *Palm job controller* to conquer by dividing successive jobs into several parallel processes to fully utilize most of the multi-core CPU resources. This multi-thread strategy accelerates ML calculations during the model selection stage and, in doing so, significantly reduces time consumption to about one sixth of the single, non-parallel process in the currently designated servers. The parallel computing strategy is also applied during the bootstrapping stage (mpi-PhyML/*Palm job controller*). However, a long running time query is possibly owing to the poor alignment of the input sequences. Users may examine the alignment of the sequences and the output tree topology, attempt to identify irrelevant sequences from the dataset and, then, resubmit the job.

The complete phylogenetic analysis workflow of PALM is a heavy computational and time-consuming task. However, we recommend incorporating contemporary models that may provide more sophisticated views on residue substitution during evolution processes. Other advanced algorithms for ML inference, *e.g.*, DPRml [36], MrBayes [37] and RAxML [15], can be integrated into PALM to provide Bayesian estimates of phylogeny and handle large phylogenetic trees in the future. Refining the entire pipeline for parallel computing is currently underway in our laboratory. Plans are also underway to introduce additional high performance computing technologies, *e.g.*, distributed computing and cloud computing, in order to mitigate the computational load of sequence alignment, likelihood estimation and bootstrapping, which is heavy along with the large amount of input data.
